# Multidimensional Bayesian adaptive testing

**DOI:** 10.3758/s13428-026-03123-9

**Published:** 2026-07-17

**Authors:** Aron Fink, Christoph König, Andreas Frey

**Affiliations:** Goethe University Frankfurt, Theodor-W.-Adorno-Platz 6, 60323 Frankfurt, Germany

**Keywords:** Multidimensional adaptive testing, Bayesian methods, Item response theory, Monte Carlo simulation

## Abstract

This paper introduces a fully Bayesian approach to multidimensional adaptive testing (MBAT). By incorporating uncertainty in both item and person parameter estimates, MBAT addresses limitations in conventional multidimensional adaptive testing (MAT), which relies on point item and person parameter estimates. A Monte Carlo simulation was conducted to evaluate the performance of MBAT compared to conventional MAT. The study was based on a four-factorial design, with the factors calibration sample size (*N* = 250, *N* = 500,* N* = 1,000), test length (*t* = 30, *t* = 60), true trait level (− 2.0, − 1.5, …, 2.0), and MAT algorithm (MAT, MBAT), across a three-dimensional trait structure with within- and between-item multidimensionality. The results showed that MBAT consistently outperformed conventional MAT in terms of the bias and mean squared error (*MSE*) of the final person parameter estimates, especially at the extremes of the trait distributions. Implemented in general-purpose software (Stan, R), the approach is computationally feasible and adaptable, providing a practical foundation for future research and applications in MAT.

## Introduction

Multidimensional adaptive testing (MAT) represents a significant advancement in the field of educational and psychological measurement, extending the principles of traditional unidimensional computerized adaptive testing (CAT; e.g., Frey, 2023) to incorporate settings where test takers’ abilities are best conceptualized across multiple interrelated latent traits (e.g., Frey & Seitz, 2009; Segall, 2010). Unlike traditional tests, which often assume a single underlying trait and administer the same fixed set of items to all test takers, MAT leverages multidimensional item response theory (MIRT; e.g., Reckase, 2016) to tailor item selection dynamically to the individuals based on their estimated position in a multidimensional latent trait space. In addition to the general gain in measurement efficiency and the alignment of standard errors across test takers by using adaptive as opposed to linear test formats (e.g., Segall, 2005), MAT has two main advantages compared to its unidimensional counterparts. Unlike unidimensional CAT, MAT allows for the simultaneous assessment of multiple latent traits, enabling a closer alignment between the theoretical structure of complex constructs and, thus, allowing a better representation of the assessment framework (e.g., Frey & Seitz, 2009; Mikolajetz & Frey, 2016). Further, by utilizing prior information about the multidimensional latent trait distribution, additional improvements in measurement efficiency can be achieved compared to that achieved by using multiple unidimensional CATs for each latent trait. This is especially true when latent traits are highly correlated (e.g., Frey & Fink, 2021; Frey & Seitz, 2011; Li & Schafer, 2005; Makransky & Glas, 2013; Paap et al., 2019; Segall, 1996; Wang & Chen, 2004).

Regardless of whether uni- or multidimensional adaptive tests are used, item selection during an adaptive test administration is driven by a statistical optimality criterion. This criterion seeks to maximize, in one way or another, the informativeness of the test with respect to the latent traits measured. In MAT, this means that, at each step of the adaptive process, the algorithm selects that one item from the pool of eligible items that most helps to determine the test taker’s position in the multidimensional latent trait space. To achieve this, item parameter estimates obtained through prior calibration studies are treated as fixed values during trait estimation and item selection. However, this approach is not entirely accurate, as it neglects the inherent uncertainty associated with these estimates. When calibration error is large—for example, when calibration sample sizes are small—ignoring this uncertainty can lead to underestimated standard errors or even biased trait estimates (e.g., Cheng et al., 2015; Doebler, 2012; Fink et al., 2025; Patton et al., 2013; van der Linden & Glas, 2000, 2001) and, therefore, jeopardizes test score interpretation.

As it is not possible to eliminate calibration error completely, statistical methods that take uncertainty in parameter estimates into account should be used. This is particularly important in adaptive testing scenarios, where uncertainty influences both the estimation of the provisional trait estimates during the test and the adaptive item selection. The issue becomes even more critical in MAT, as uncertainty in item parameter estimates affects multiple trait dimensions simultaneously. Errors in one dimension can influence item selection and trait estimation across other dimensions due to the interdependence of traits in MIRT models.

One promising approach to address these challenges is the use of Bayesian methods. By treating both item parameter and trait estimates as random variables with associated probability distributions, Bayesian methods naturally incorporate uncertainty into the estimation process. For this purpose, van der Linden and Ren (2020) proposed a fully Bayesian algorithm for unidimensional CATs with dichotomously scored items, which was later extended to polytomous items by Ren et al. (2020). Both studies found that the Bayesian algorithm produced more reliable standard error estimates across different trait levels than conventional CAT, particularly in the lower range of the trait distribution. Niu and Choi (2022), who extended this approach and presented a more efficient version of the integrated Gibbs sampler, corroborated the previous findings. Recently, Fink et al. (2025) proposed a refined version of Niu and Choi’s algorithm by using the no-U-turn sampler (NUTS; Hoffman & Gelman, 2014), an adaptive version of the Hamiltonian Monte Carlo algorithm (HMC; Neal, 2011), which is implemented in Stan (Stan Development Team, 2023). This version allows efficient sampling without requiring costly tuning runs and the hand-tuning of sampler parameters. They compared the algorithm with traditional non-Bayesian CAT procedures as well as with two other approaches to account for uncertainty in parameter estimates during CAT that are based on measurement error modeling. Their simulation study showed that the fully Bayesian approach clearly outperformed the other approaches in terms of bias and mean squared error (*MSE*) reduction, especially when the calibration error was high (Fink et al., 2025).

However, each of the aforementioned studies only referred to the unidimensional case. The primary aim of this study was, therefore, to introduce a multidimensional extension of the fully Bayesian adaptive testing algorithm and to investigate its performance in comparison to more traditional MAT approaches. The approach was implemented in general-purpose software for Bayesian analyses (Stan); the code to run the approach is provided in the electronic supplementary material (ESM) of this paper and can therefore be easily used and extended by researchers and practitioners.

In the following sections, we first provide a brief description of MAT methods and a short introduction to Bayesian adaptive testing. Based on these descriptions, the algorithm for multidimensional Bayesian adaptive testing (MBAT) is then derived. Finally, we present results from a Monte Carlo simulation study that investigated how well MBAT performed compared to conventional MAT algorithms, and we conclude with a discussion.

### Multidimensional adaptive testing

MAT extends the principles of CAT to assessments that measure multiple latent traits simultaneously. Like its unidimensional counterpart, MAT aims to tailor item selection to the individual response behavior. With the use of MIRT models, MAT selects items that are most informative for an individual’s trait profile. A general MIRT model for dichotomously scored items is the multidimensional compensatory three-parameter logistic (M3PL; Reckase, 2016) model. The M3PL model specifies the probability that an individual test taker j=1,⋯,N with a *p*-dimensional trait vector ${{\boldsymbol{\uptheta}}}_{j}=({\theta}_{1j},\dots ,{\theta}_{pj})$ will answer item *i* correctly (${u}_{ji}=1$) as

1 $$P\left({u}_{ji}=1|{{\boldsymbol{\uptheta}}}_{j},{\mathbf{a}}_{i},{{c}_{i},d}_{i}\right)={c}_{i}+\left(1-{c}_{i}\right)\frac{\mathrm{exp}\left({{{\boldsymbol{a}}}_{i}}^{\mathrm{T}}{{\boldsymbol{\uptheta}}}_{j}+{d}_{i}\right)}{1+\mathrm{exp}\left({{{\boldsymbol{a}}}_{i}}^{\mathrm{T}}{{\boldsymbol{\uptheta}}}_{j}+{d}_{i}\right)}.$$

The relationship between item *i* and the latent trait dimensions is captured by the *p-*dimensional item loading vector ${\mathbf{a}}_{i}$. In cases of between-item multidimensionality, only one element of ${\mathbf{a}}_{i}$ is nonzero, indicating that the item loads on one single dimension. In contrast, within-item multidimensionality is characterized by multiple nonzero elements in the vector ${\mathbf{a}}_{i}$, reflecting that the item measures several of the *p* dimensions. The ${d}_{i}$ parameter is a scalar value that is related to item difficulty (see Eq. 2), and the pseudoguessing parameter ${c}_{i}$ accounts for the lower asymptote of the item response function, modeling the likelihood of correct responses due to random guessing. The multidimensional item difficulty *MDIFF*, which is the distance from the origin of the item response surface to the point of steepest slope in the direction in which the item provides the most information, can be calculated as

2 $$MDIFF=\frac{-{d}_{i}}{\sqrt{{\sum}_{k=1}^{p}{\mathbf{a}}_{ik}^{2}}}.$$

The denominator of this equation is the multidimensional discrimination or *MDISC* of item *i*. The model is compensatory in that an examinee with a high value on one dimension can compensate for a low value on another, which results in this examinee having the same probability of a correct response as another examinee whose abilities are more evenly distributed across the dimensions (for partial or noncompensatory MIRT models, see, e.g., Reckase, 2016). The M2PL model, which was used in this study, can be derived from the M3PL by setting all ${c}_{i}$ parameters to zero.

In order to select the next item to be administered during MAT based on the provisional estimate of a test taker’s position in the multidimensional trait space, both maximum likelihood and Bayesian approaches can be used. Of these, the Bayesian approach proposed by Segall (1996) is probably the most popular. It has demonstrated strong performance and robustness, offering high accuracy and precision in trait estimation across a wide range of MAT configurations (Mulder & van der Linden, 2009; Veldkamp & van der Linden, 2002; Wang & Chang, 2011; Wang et al., 2011). Segall’s item selection method acknowledges that responses to items targeting specific dimensions also yield information about other correlated dimensions. To optimize item selection, it incorporates the *p* × *p*-dimensional variance–covariance matrix **Ф** of the latent traits as prior knowledge about the interrelationships among the measured dimensions. Throughout the testing process, items that maximize the overall gain in measurement precision across all dimensions are selected from the pool of remaining items, using the *D*-optimality criterion as the item selection criterion. The criterion for candidate item ${i}^{*}$ after answering an item set *T* is calculated as

3 $${D}_{T+{i}^{*}}=\left|{\mathbf{I}}_{T}\left({\widehat{{\boldsymbol{\uptheta}}}}_{j}\right)+{\mathbf{I}}_{{i}^{*}}\left({\widehat{{\boldsymbol{\uptheta}}}}_{j},{{\boldsymbol{\upxi}}}_{{i}^{*}}\right)+{{\boldsymbol{\Phi}}}^{-1}\right|,$$

where ${\mathbf{I}}_{T}\left({\widehat{{\boldsymbol{\uptheta}}}}_{j}\right)={\sum}_{i\in T}{\mathbf{I}}_{i}\left({\widehat{{\boldsymbol{\uptheta}}}}_{j},{{\boldsymbol{\upxi}}}_{T}\right)$ is the test information matrix of item set *T* with item parameters ${{\boldsymbol{\upxi}}}_{T}$ in the estimated position ${\widehat{{\boldsymbol{\uptheta}}}}_{j}$ of test taker *j* in the multidimensional latent trait space, calculated by summing up the item information matrices of all already answered items ${\mathbf{I}}_{i}\left({\widehat{{\boldsymbol{\uptheta}}}}_{\mathrm{j}}\right)=\left[{1-P}_{i}\left({\widehat{{\boldsymbol{\uptheta}}}}_{j}\right)\right]{P}_{i}({\widehat{{\boldsymbol{\uptheta}}}}_{j}){\mathbf{a}}_{i}{{\mathbf{a}}_{\mathrm{i}}}^{T}$*,* and ${\mathbf{I}}_{{i}^{*}}\left({\widehat{{\boldsymbol{\uptheta}}}}_{j},{{\boldsymbol{\upxi}}}_{{i}^{*}}\right)$ is the item information matrix of candidate item ${i}^{*}$ with item parameters ${{\boldsymbol{\upxi}}}_{{i}^{*}}\equiv ({\mathbf{a}}_{i}, {d}_{i})$*.* The next item to be administered ${i}_{t+1}$ is selected from the pool of remaining items by

4 $${i}_{t+1}=\mathrm{a}\mathrm{r}\mathrm{g}\;\underset{i\in {R}_{t+1}}{\mathrm{m}\mathrm{a}\mathrm{x}}{D}_{T+{i}^{*}}.$$

To estimate ${\widehat{{\boldsymbol{\uptheta}}}}_{j}$, the multidimensional maximum a posteriori (MAP) estimator can be used, incorporating the same prior information **Ф** (Segall, 1996). As MAT progresses, at each step, the next item is selected based on its potential to yield the greatest reduction in the volume of the credibility ellipsoid around the current estimate of the test taker’s latent trait vector.

### Bayesian adaptive testing

As described in the introduction, the standard approach to CAT and MAT uses point estimates for the latent trait as well as for the item parameter estimates. This disregards the fact that those estimates contain a certain degree of error. The fully Bayesian approach to adaptive testing, introduced by van der Linden and Ren (2020) and later extended in a number of studies (Fink et al., 2025; Niu & Choi, 2022; Ren et al., 2020), takes this uncertainty into account. In a unidimensional Bayesian CAT (BAT), the posterior distribution of ${\theta}_{j}$ after answering the *t*th item of a test can be defined as

5 $$f\left({\theta}_{j}|{\mathrm{u}}_{t}\right)=\frac{\int f\left({u}_{t}|{\theta}_{j},{{\boldsymbol{\upxi}}}_{{i}_{t}}\right)f\left({\theta}_{j}|{u}_{t-1}\right)f\left({{\boldsymbol{\upxi}}}_{{i}_{t}}\right)d{{\boldsymbol{\upxi}}}_{{i}_{t}}}{\iint f\left({u}_{t}|{\theta}_{j},{{\boldsymbol{\upxi}}}_{{i}_{t}}\right)f\left({\theta}_{j}|{u}_{t-1}\right)f\left({{\boldsymbol{\upxi}}}_{{i}_{t}}\right)d{\theta}_{j}d{{\boldsymbol{\upxi}}}_{{i}_{t}}}.$$

Here, $f\left({u}_{t}|{\theta}_{j},{{\boldsymbol{\upxi}}}_{{i}_{t}}\right)$ denotes the likelihood of observing response ${u}_{t}$ to item ${i}_{t}$ conditional on ${\theta}_{j}$ and the corresponding set of item parameters ${{\boldsymbol{\upxi}}}_{{\boldsymbol{i}}}$. Prior distributions of the item and ability parameters are reflected by $f\left({{\boldsymbol{\upxi}}}_{{i}_{t}}\right)$ and $f\left({\theta}_{j}|{u}_{t-1}\right)$. As CAT progresses, the ability distribution is iteratively refined with each new item response. In this process, the posterior distribution $f\left({\theta}_{j}|{u}_{t}\right)$ obtained after the *t*th response becomes the prior for estimating ${\theta}_{j}$ when administering the (*t* + 1)th item. Because the Markov chain Monte Carlo (MCMC) methods used for Bayesian analysis have traditionally been seen as too slow for real-time use in CAT, van der Linden and Ren (2020) proposed a fully Bayesian adaptive testing (BAT) algorithm that resolved, this issue. The basic functionality of the algorithm is as follows (see van der Linden & Ren, 2020, for a detailed description):

Having an item pool that has been precalibrated using an MCMC algorithm, the vectors ${{\boldsymbol{\upxi}}}_{i}=({{\boldsymbol{\upxi}}}_{i}^{\left(1\right)},\dots ,{{\boldsymbol{\upxi}}}_{i}^{\left(S\right)}$) represent *s* = 1, …, *S* stored post-burn-in samples of item *i*’s parameter estimates. During the operational CAT phase, when the *t*th item is answered, samples are drawn from ${{\boldsymbol{\upxi}}}_{{i}_{t}}$​​ for the posterior update of ${\theta}_{j}$. After each update, a new set of post-burn-in draws (${\theta}_{t}^{\left(1\right)},\dots ,{\theta}_{t}^{\left(S\right)}$) is stored, replacing the previous draws from the (t-1) th step. These updated draws are then used to define an empirical prior for the next iteration as ${\theta}_{t}\sim N({\widehat{\upmu }}_{t-1},{\widehat{\upsigma }}_{t-1}^{2})$, where

6 $${\widehat{\upmu }}_{t-1}={S}^{-1}\sum\nolimits_{s=1}^{S}{\theta}_{t-1}^{\left(s\right)},$$

and

7 $${\widehat{\upsigma }}_{t-1}^{2}={S}^{-1}\sum\nolimits_{s=1}^{S}\left({\uptheta}_{\mathrm{t}-1}^{\left(\mathrm{s}\right)}-{\widehat{\upmu }}_{t-1}\right).$$

To further mitigate the impact of calibration error, it is essential to also incorporate uncertainty into item and person parameter estimates during adaptive item selection. Using the stored draws for item and person parameters mentioned above, van der Linden and Ren (2020) described a Bayesian version of the traditional maximum information criterion as

8 $${i}_{t+1}=\mathrm{arg}\;\underset{i\in {R}_{t+1}}{\mathrm{max}}\left\{{S}^{-1}\sum\nolimits_{s=1}^{S}{I}_{i}({\theta}_{j}^{(s)}; \;{{\boldsymbol{\upxi}}}_{{i}_{t}}^{(s)})\right\},$$

which is simply the average item information for candidate items across permanently stored posterior draws for person and item parameters.

### Multidimensional Bayesian adaptive testing

To generalize the idea of unidimensional BAT to the multidimensional case, the vector ${{\boldsymbol{\upxi}}}_{i}$ contains *S p*-dimensional draws for ${\mathbf{a}}_{i}$, as well as *S* draws for ${d}_{i}$, saved from the calibration step. For the theta update after each respective response, the normal prior that is used in unidimensional BAT is replaced with a multivariate normal distribution ${{\boldsymbol{\uptheta}}}_{t}\sim \mathrm{M}\mathrm{V}\mathrm{N}({\widehat{{\boldsymbol{\upmu}}}}_{t-1},{\widehat{{\boldsymbol{\Phi}}}}_{t-1})$, with the *p*-dimensional vector of means ${\widehat{{\boldsymbol{\upmu}}}}_{t-1}$ and the *p* × *p*-dimensional variance–covariance matrix ${\widehat{{\boldsymbol{\Phi}}}}_{t-1}$, both calculated from the saved posterior draws from the last update of ${{\boldsymbol{\uptheta}}}_{t-1}$ as

9 $${\widehat{{\boldsymbol{\upmu}}}}_{t-1}={S}^{-1}\sum\nolimits_{s=1}^{S}{{\boldsymbol{\uptheta}}}_{t-1}^{\left(s\right)},$$

and

10 $${\widehat{{\boldsymbol{\Phi}}}}_{t-1}=\left[\begin{array}{ccc}Var\left({{\boldsymbol{\uptheta}}}_{1,t-1}^{(s)}\right)& \dots & cov({{\boldsymbol{\uptheta}}}_{1,t-1}^{(s)},{{\boldsymbol{\uptheta}}}_{p,t-1}^{(s)})\\ \begin{array}{c}\vdots \\ cov({{\boldsymbol{\uptheta}}}_{p,t-1}^{(s)},{{\boldsymbol{\uptheta}}}_{1,t-1}^{(s)})\end{array}& \begin{array}{c}\ddots \\ \dots \end{array}& \begin{array}{c}\vdots \\ Var\left({{\boldsymbol{\uptheta}}}_{p,t-1}^{(s)}\right)\end{array}\end{array}\right].$$

As in the unidimensional case, the posterior distribution of the trait after the *t*th item is used as an empirical prior for the estimation after the (t+1) th item and, therefore, contains the complete response history of the test taker.

Following this, the fully Bayesian version of the *D*-optimality item selection criterion outlined in Eq. 4 can be derived by averaging the determinant *D* across the saved posterior draws for the item and person parameters, so that the next item from the pool is selected by

11 $${i}_{t+1}=\mathrm{arg}\;\mathrm{max}\left\{{S}^{-1}\sum\nolimits_{s=1}^{S}{D}_{T+{i}^{*}}({{\boldsymbol{\uptheta}}}_{j}^{(s)},{{\boldsymbol{\upxi}}}_{i}^{\left(s\right)})\right\}.$$

### Research questions

The primary aim of this study was to evaluate the performance of MBAT with regard to the quality of the resulting trait estimates compared to more traditional approaches to MAT. Therefore, the first research question (RQ1) was posed: How does MBAT perform with regard to measurement precision and the bias of trait estimates compared to conventional MAT methods?

A key factor likely to affect the performance of the method is the magnitude of the calibration error present, which is closely tied to the size of the calibration sample. This led to the second research question (RQ2): Does the relative performance of MBAT and conventional MAT methods vary depending on the calibration sample size?

Additionally, the item selection ratio, defined as the proportion of the item pool used during a test, affects how calibration error impacts trait estimation. A lower item selection ratio, which is associated with shorter test lengths, increases the risk of selecting poorly estimated items (van der Linden & Ren, 2020), prompting the third research question (RQ3): Does test length influence the differences in performance between MBAT and conventional MAT methods in the presence of calibration error?

## Method

To answer the aforementioned research questions, a Monte Carlo simulation was carried out. The ESM, including the code to run the MBAT, is available in an OSF repository, which can be retrieved from https://osf.io/43nxm/?view_only=bf1af24dab2046f295a599d0bb10b011.

The simulation was based on a fully crossed four-factorial design with three between-factors and one within-factor. First, the *calibration sample size* was manipulated across three levels (*N* = 250, *N* = 500,* N* = 1,000). This factor represents the number of responses per item in the calibration and, therefore, reflects differing degrees of item parameter uncertainty. Second, the *test length* was varied at two levels (*t* = 30, *t* = 60), allowing the investigation of how the number of administered items affected the quality of trait estimation. While the number of items in the item pool was kept constant at 300, this factor also reflects the item selection ratio, namely, 10:1 and 5:1. Third, the *MAT algorithm* used was varied in order to compare the two conditions (MAT, MBAT). The first one reflected the conventional approach to MAT in which item parameters are treated as fixed (ignoring calibration error) and point estimates for the person parameters are used for item selection. The second one was the new fully Bayesian MAT approach (MBAT) described above, which explicitly incorporates uncertainty in item and person parameter estimates into the adaptive process. Fourth, the *true trait level*s of the test takers were varied systematically. For each simulation, true values per dimension were drawn from a discrete set of nine levels ranging from − 2.0 to 2.0 in increments of 0.5 (i.e., ${\theta}_{p}$ = − 2.0, − 1.5, …, 2.0), yielding a broad range of latent trait levels with which the estimation quality could be estimated. The number of simulated dimensions was fixed at 3, which means that each test taker was assigned three trait values. Although these values were sampled from predefined levels, they were generated to maintain the intended correlation structure among dimensions, ensuring realistic interdependencies between the traits. Moreover, the data were simulated such that each trait level in each dimension was represented by *N* = 250 test takers. This design guaranteed equal representation across all ability levels and dimensions, making it possible to directly compare bias and the *MSE* without confounding effects due to unequal group sizes.

### Simulation procedure

To demonstrate the flexibility of the proposed approach, we simulated an item pool that exhibited a mixture of within-item and between-item multidimensionality. The goal was not to systematically compare these structures, but to evaluate performance in a heterogeneous pool resembling operational testing scenarios, where both item types are represented. We used the M2PL model in this simulation. For each item, three *a*-parameters were independently drawn from a log-normal distribution ${a}_{p,i}\sim \mathrm{l}\mathrm{o}\mathrm{g}N\left(0.2, 0.3\right)$. Subsequently, a subset of the loadings was randomly set to zero, resulting in items that loaded on one, two, or all three dimensions. This process yielded a pool in which each dimension was represented by 155 items in total. Specifically, 25, 50, and 75 items loaded exclusively on Dimensions 1, 2, and 3, respectively. In addition, 70 items loaded on both Dimensions 1 and 2, 20 items on Dimensions 1 and 3, and 45 items on Dimensions 2 and 3. Finally, 15 items loaded on all three dimensions. Thus, the pool contained 150 unidimensional items and 150 multidimensional items, resulting in a balanced mixture of between-item and within-item multidimensionality. This heterogeneous structure was chosen to illustrate that the proposed approach can accommodate item pools with varying dimensional representation and structural complexity, as often observed in operational testing scenarios. The *d*-parameters were drawn from a standard normal distribution, ${d}_{i}\sim N\left(\mathrm{0,1}\right)$.

After simulating the item pool, the simulation proceeded in two distinct stages: (1) an item calibration stage and (2) an adaptive testing stage. Different data-generating approaches for person parameters were used in these two stages to reflect their different purposes.

In the first stage, the calibration was simulated. To this end, *N* responses per item were simulated according to the levels of the calibration sample size factor, following the M2PL model. The simulated responses were based on the item parameters obtained from the step described above as well as on the person parameters sampled from a multivariate normal distribution ${\boldsymbol{\uptheta}}\sim \mathrm{M}\mathrm{V}\mathrm{N}\left({\boldsymbol{\upmu}},{\boldsymbol{\Phi}}\right),$ with ${\boldsymbol{\upmu}}=(\mathrm{0,0,0})$ and $${\boldsymbol{\Phi}}=\left[\begin{array}{ccc}1& .50& .50\\ .50& 1& .50\\ .50& .50& 1\end{array}\right]$$. This stage served solely to obtain realistic item parameter estimates and corresponding calibration uncertainty. We used a hierarchical model to estimate the M2PL model in Stan (see ESM C1 for the respective Stan file). The prior distributions used in this model reflect standard practice for these kinds of IRT models. For the item loadings ${a}_{p,i}$, a log-normal prior ${a}_{p}\sim \mathrm{l}\mathrm{o}\mathrm{g}N\left({\upmu}_{{a}_{p}}, 1\right)$ was used where the location parameter ${\upmu}_{{a}_{p}}$ was assigned a hyperprior ${\upmu}_{{a}_{p}}\sim \mathrm{C}\mathrm{a}\mathrm{u}\mathrm{c}\mathrm{h}\mathrm{y}(\mathrm{0,0.5})$. The log-normal distribution ensures positive and right-skewed discrimination parameters. The Cauchy-distributed hyperprior, centered at 0 with scale 0.5, provides weakly informative regularization while allowing occasional larger values of ${\upmu}_{{a}_{p}}$ and thus potentially larger discriminations. The item d-parameters were assigned a normal prior $d\sim N\left({\upmu}_{d},{\upsigma}_{d}\right)$, with mean ${\upmu}_{d}\sim \mathrm{C}\mathrm{a}\mathrm{u}\mathrm{c}\mathrm{h}\mathrm{y}(\mathrm{0,2})$ and standard deviation ${\upsigma}_{d}\sim \mathrm{C}\mathrm{a}\mathrm{u}\mathrm{c}\mathrm{h}\mathrm{y}(\mathrm{0,1})$. The normal distribution allows both positive and negative difficulty values centered around ${\upmu}_{d}$, while the Cauchy-distributed hyperpriors provide weakly informative regularization, allowing substantial variability in both the location and scale parameters of the normal distribution when supported by the data. Examinee abilities ${\boldsymbol{\uptheta}}$ were modeled using a multivariate normal distribution ${\boldsymbol{\uptheta}}\sim \mathrm{M}\mathrm{V}\mathrm{N}\left({\boldsymbol{\upmu}},{\boldsymbol{\Phi}}\right),$ with ${\boldsymbol{\upmu}}=(\mathrm{0,0,0})$ and ${\boldsymbol{\Phi}}\sim \mathrm{L}\mathrm{K}\mathrm{J}\mathrm{c}\mathrm{o}\mathrm{r}\mathrm{r}(1)$. The Lewandowski–Kurowicka–Joe (LKJ) prior with a shape parameter of 1 assumes a uniform prior across possible correlation matrices, thereby avoiding strong assumptions about intertrait relationships. Four chains, each with a length of 1,500 with 500 burn-in draws, were set up. Each of the *S* = 4,000 post-burn-in draws were saved for later use during MBAT.

In the second stage, adaptive tests were simulated using the calibrated item parameters from stage 1. In contrast to the calibration stage, true trait levels were drawn from the previously described discrete set of nine values. To ensure balanced representation, each level occurred exactly 250 times per dimension. Given the three dimensions and the nine trait levels, this resulted in a total sample size of *N* = 2,250 simulated test takers. Trait values were generated such that the correlation between each pair of the three dimensions across all simulated test takers was equal to .5. For this we used the procedure described in Ferrari and Barbiero (2012) which allows for simulating discrete values with a prespecified correlation structure via an underlying multivariate normal distribution and subsequent discretization. Because discretization can alter the resulting correlations, we verified the empirical correlations among the discretized theta values. These correlations closely approximated the intended value of .5 (mean correlations across replications, ${r}_{12}=.48$, ${r}_{13}=.47$, ${r}_{23}=.48$). The value of .5 was chosen to represent a moderate association between dimensions. Other target correlation structures can also be specified using the same procedure; however, the degree to which the resulting discretized variables reproduce the target correlations depends on both the chosen correlation structure itself and the marginal distributions and discretization scheme, and should therefore be evaluated for the specific application.

For the MAT condition, we used the MAP estimator for trait estimation after each response during the adaptive test and the *D*-optimality criterion for item selection, reflecting common practice in multidimensional adaptive testing. Trait estimation and item selection were based on the point estimates for the item parameters that stemmed from the calibration step (i.e., the means of the posterior distributions). The prior used for ability estimation via MAP and item selection via the *D*-optimality criterion was set to its respective estimate from the calibration step. For the MBAT condition, the prior for the person parameter for the first response was set to ${\boldsymbol{\uptheta}}\sim \mathrm{M}\mathrm{V}\mathrm{N}\left({\boldsymbol{\upmu}},\widehat{{\boldsymbol{\Phi}}}\right),$ with ${\boldsymbol{\upmu}}=(\mathrm{0,0,0})$ and $\widehat{{\boldsymbol{\Phi}}}$ reflecting the estimated correlation matrix from the calibration step. For the subsequent item responses, the prior of ${{\boldsymbol{\uptheta}}}_{j}$ after the *t*th response was set to the posterior after the (t-1) th response. For the theta update, item parameters ${\mathbf{a}}_{i}$ and ${d}_{i}$ were resampled from the *S* = 4,000 permanently stored posterior draws from the calibration step. For each person’s parameter update, four chains, each with a length of 500 with 250 burn-in draws, were set up. Convergence was reached for each simulation run, which was indicated by the *R*-hat diagnostic (Vehtari et al., 2020) of *R* < 1.02. All post-burn-in draws of the estimation of the provisional ability parameter were initially stored for subsequent use in the adaptive item selection. For the item selection, 400 draws were resampled from the stored draws of the item parameters as well as the person parameters in each step of the adaptive test in order to calculate the item selection criterion in Eq. 11. The final ability estimate corresponded to the posterior mean of the joint posterior distribution of ${{\boldsymbol{\uptheta}}}_{j}.$

The whole simulation procedure was replicated *R* = 20 times. The number of replications (R=20) was determined based on preliminary analyses, indicating that the resulting Monte Carlo standard errors would be sufficiently small while keeping computational costs at a reasonable level. In the final analysis, the average Monte Carlo standard error across all conditions was 0.013 for bias and 0.005 for *MSE* (with maximum values of 0.023 and 0.016). The simulation was conducted in R (R Core Team, 2023) using mirtCAT (Chalmers, 2016), mirt (Chalmers, 2012), Stan (Stan Development Team, 2023), and the cmdstanr package (Gabry et al., 2025).

### Evaluation criteria

The evaluation criteria for the quality of the dimension-specific ${\widehat{\theta }}_{p}$ estimates were the bias and the *MSE* of ${\widehat{\theta }}_{p}$, each conditional on the true ability level ${\theta}_{p}$. The conditional bias at point ${\theta}_{p}$ on the trait scale of dimension *p* was calculated as

12 $${\mathrm{B}\mathrm{i}\mathrm{a}\mathrm{s}}_{ {\theta}_{p}}= \frac{1}{R\cdot N}{\sum}_{r=1}^{R}{\sum}_{j=1}^{N}({\widehat{\theta }}_{pjr} -{\theta}_{pjr}),$$

and the *MSE*, conditional on ${\theta}_{p},$ as

13 $${MSE}_{ {\uptheta}_{p}}= \frac{1}{R\cdot N}{\sum}_{r=1}^{R}{\sum}_{j=1}^{N}({\widehat{\theta }}_{pjr} -{\theta}_{pjr}{)}^{2},$$

where j=1,⋯,N indexes simulees within each replication, and r=1,⋯,R indexes simulation replications.

## Results

**Fig. 1:**
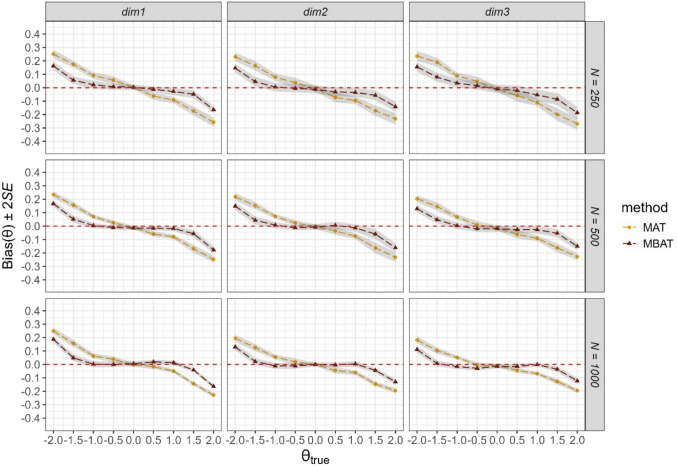
Conditional bias of person parameter estimates for the two simulated multidimensional adaptive testing approaches for three dimensions, three calibration sample sizes *N*, and a test length of *t* = 60. *Note.* Shaded areas indicate ± 2 *SE*. The *SE* represents Monte Carlo error based on variability across replications

**Fig. 2:**
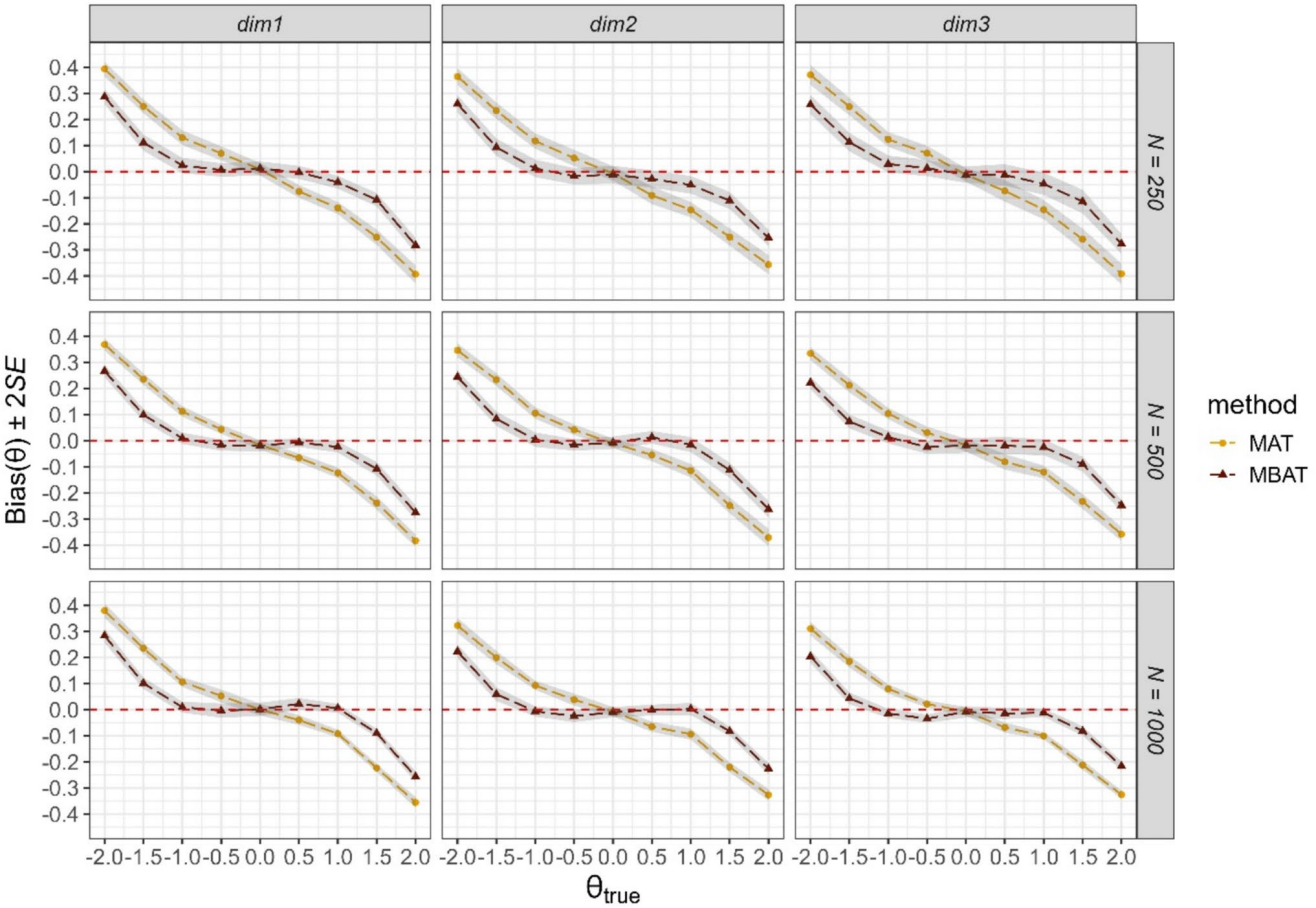
Conditional bias of person parameter estimates for the two simulated multidimensional adaptive testing approaches for three dimensions, three calibration sample sizes *N*, and a test length of *t* = 30. *Note.* Shaded areas indicate ± 2 *SE*. The *SE* represents Monte Carlo error based on variability across replications

The results for the conditional bias for the nine ability levels across the measured dimensions and a test length of *t* = 60 items are depicted in Fig. 1 (for the numerical results, see ESM Table M1). The standard MAT condition resulted in substantial bias in the ability estimates across all dimensions and conditions, exhibiting the well-known shrinkage effect associated with Bayesian estimation in IRT models, where low trait levels tend to be overestimated and high trait levels underestimated. In contrast, the MBAT approach effectively mitigated this bias across the full range of trait levels. For all dimensions and calibration sample sizes, MBAT consistently produced lower bias values, with the greatest improvements observed at the extremes of the trait distribution. Notably, the calibration sample size appeared to have a minimal influence on the size of the conditional bias. A similar pattern emerged for a test length of *t* = 30 items (Fig. 2). As expected, bias values were generally higher than those obtained with the longer test length of 60 items. With the exception of the medium trait level (*θ* = 0), the standard MAT condition produced notably biased estimates.

**Fig. 3:**
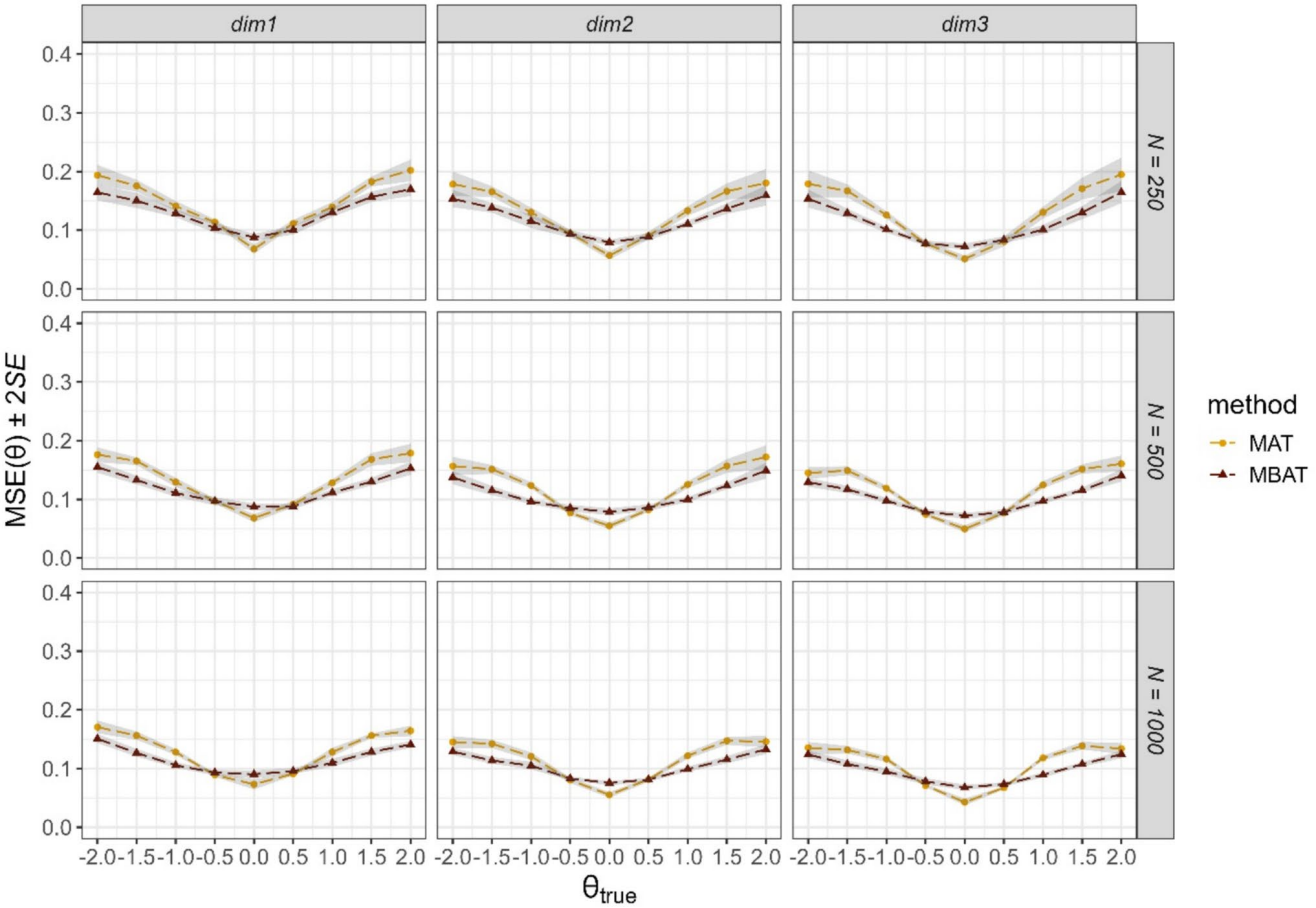
Conditional mean squared errors of person parameter estimates for the two simulated multidimensional adaptive testing approaches for three dimensions, three calibration sample sizes *N*, and a test length of *t* = 60*. Note.* Shaded areas indicate ± 2 *SE*. The *SE* represents Monte Carlo error based on variability across replications

**Fig. 4:**
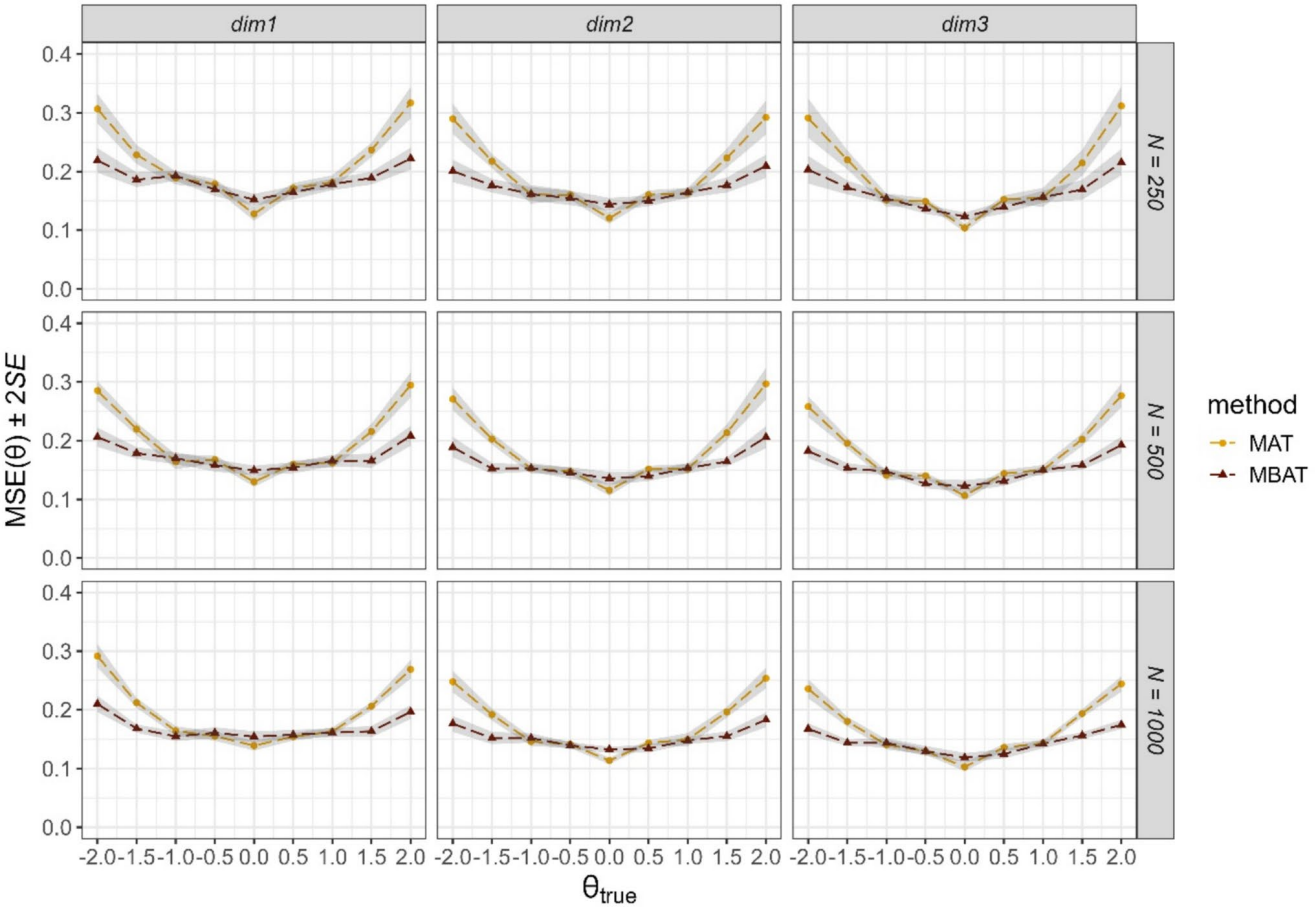
Conditional mean squared errors of person parameter estimates for the two simulated multidimensional adaptive testing approaches for three dimensions, three calibration sample sizes *N*, and a test length of *t* = 30. *Note.* Shaded areas indicate ± 2 *SE*. The *SE* represents Monte Carlo error based on variability across replications

The results regarding the conditional *MSE* of the final trait estimates are depicted in Fig. 3 (*t* = 60) and Fig. 4 (*t* = 30; for the numerical results, see ESM Table M2). Although some bias remained at the extremes of the trait distribution when using MBAT, the MBAT approach consistently reduced the magnitude of bias across all conditions, even for this relatively short test length of 30 items, which simultaneously assessed three dimensions. The results are presented separately for each of the three dimensions and across three calibration sample sizes (*N* = 250, *N* = 500, *N* = 1,000).

Across most conditions, MBAT outperformed standard MAT in terms of the precision of the final trait estimates, as reflected in lower *MSE* values. This advantage was particularly pronounced at the extremes of the trait continuum and for *t* = 30, where MAT showed markedly higher *MSE* values when calibration error was not accounted for. In contrast, MBAT effectively reduced the *MSE.* At a medium trait level of *θ* = 0, MAT resulted in slightly lower *MSE* values across all conditions. The calibration sample size had a general effect on estimation precision for both methods. As the sample size increased from 250 to 1,000, the *MSE* values decreased, reflecting more accurate item parameter estimates and, therefore, less calibration error. However, even at the smallest calibration sample size (*N* = 250), MBAT showed clear improvements in comparison to MAT, especially in the marginal regions of the trait distribution. At *N* = 1,000, while the difference between the two methods narrowed slightly at the center of the trait distribution, MBAT continued to yield better performance at the tails. These findings were consistent across all three dimensions.

When comparing the two different test lengths, as expected, the overall *MSE* values were higher for *t* = 30 than for the longer test length of 60 items. Nevertheless, the general

pattern was that MBAT consistently yielded lower *MSE* values than MAT across nearly all trait levels, dimensions, and calibration sample sizes. This advantage was especially pronounced at the extremes of the trait distribution.

## Discussion

The present study introduced and evaluated a fully Bayesian approach to MAT, extending previous work on unidimensional BAT to the multidimensional context. By means of a simulation study, we compared the MBAT approach with a conventional MAT approach that ignores item calibration error and relies on point estimates of item and person parameters.

The results provide evidence that MBAT offers substantial improvements regarding the precision and accuracy of the trait estimates. Across all simulated conditions, MBAT consistently outperformed the conventional MAT algorithm with respect to both the bias and the *MSE* of the resulting trait estimates (RQ1). The advantage was most pronounced at the extremes of the latent trait distribution. This result is in line with previous studies regarding the performance of unidimensional fully Bayesian adaptive tests compared to approaches that ignore parameter uncertainty (e.g., Fink et al., 2025; Niu & Choi, 2022; van der Linden & Ren, 2020).

Furthermore, and with regard to RQ2, MBAT demonstrated robust performance even when the calibration sample sizes were rather small and, therefore, the impact of item calibration error was larger. While both methods benefited from larger calibration samples, MBAT maintained its advantage across all simulated sample sizes, highlighting its capacity to compensate for increased uncertainty during item calibration. This makes the approach also suitable for test scenarios with continuous item pool calibrations, where the parameter uncertainty is rather high at the beginning of the approach, due to rather small samples, and decreases over time through the continuous update of the parameter estimates (e.g., Fink et al., 2018; Frey & Fink, 2025).

Finally, this study also examined the role of test length on the results (RQ3), revealing that, while shorter tests resulted in greater overall estimation errors, MBAT still provided improvements over the conventional approach to MAT. Even with a relatively short test length of 30 items in a three-dimensional testing context, MBAT substantially reduced both bias and the *MSE* compared to conventional MAT. This finding highlights the robustness of MBAT and shows that its ability to account for parameter uncertainty remains effective even under constrained testing conditions, such as limited testing time and high-dimensional assessments (see, e.g., Frey & Fink, 2021).

Thus, the results of our study contribute to the growing body of evidence that supports fully Bayesian approaches in adaptive testing. In addition to its psychometric advantages, MBAT can be implemented using widely accessible software (Stan and R). We have made the full estimation and item selection algorithm publicly available so that it can be used by practitioners and researchers. This makes MBAT not only a psychometrically sound but also a practically feasible alternative to existing MAT procedures. Moreover, the use of a general-purpose probabilistic programming framework such as Stan enables high flexibility, allowing users to extend our approach to other models, such as polytomous response models or partial and noncompensatory MIRT models. It also makes it possible to easily specify different prior specifications. While the multivariate normal priors we used for the onset of the adaptive test and for the continuous Bayesian updating of ability estimates, with empirical means and covariance matrices derived from the previous posterior distribution, reflect a computationally efficient and widely accepted approach, other assumptions are also reasonable. In more specialized contexts, in which prior information (e.g., domain knowledge, historical test data, known population heterogeneity) suggests nonnormality or reduced variance in certain trait dimensions, other prior formulations may be beneficial. Possible alternatives include multivariate skew-normal distributions or even hierarchical priors that incorporate group-level structures, enabling a more tailored modeling of subpopulation differences. However, these alternatives come at the cost of increased computational complexity, which must be weighed against their potential gains in estimation accuracy. This is particularly true in multidimensional settings, where the computational burden increases along with the number of trait dimensions. Further research is needed to evaluate the practical trade-offs between flexibility in prior specification and the computational feasibility required for operational testing scenarios. The present paper provides a versatile foundation for these possible future developments in MBAT.

Beyond educational testing, MBAT is also highly relevant for experimental and cognitive research, where reliable and unbiased measurement is essential for drawing valid conclusions. Many studies assess multiple related constructs simultaneously. MBAT enables efficient measurement of these constructs while explicitly accounting for their intercorrelations and thus improving estimation accuracy. Further, in intervention studies, more accurate and less biased ability estimates reduce the risk of attributing observed effects to experimental manipulations when they may instead reflect measurement error. By improving precision, quantifying uncertainty, and reducing participant burden through shorter testing times, MBAT also supports the design of robust and efficient research designs.

Nevertheless, some limitations should be acknowledged. The simulation focused on a specific MIRT model (M2PL) and was limited to a three-dimensional test with a fixed covariance matrix. As such, the results may not fully generalize to other multidimensional configurations, higher-dimensional trait spaces, varying intertrait correlations, or alternative proportions of within- and between-item multidimensionality. However, given that the simulation design relied on a simple yet representative multidimensional structure, including a mixture of within- and between-item multidimensionality, it is reasonable to expect that the observed advantages of MBAT would extend to other dimensional structures, loading configurations, and covariance patterns. Future research should empirically confirm this assumption by applying MBAT across a wider range of scenarios.

Furthermore, although the efficiency of MBAT was demonstrated to be feasible for simulation purposes, a real-time application in operational test settings remains a potential challenge, particularly for high-dimensional assessments. In the present simulation, the average running time per adaptive step was around 0.4 s, suggesting that the approach is feasible for real-time implementation under the studied conditions. However, computational demands are expected to increase with the number of dimensions and additional operational constraints such as content balancing or exposure control. Future research should therefore explore the implementation of MBAT in practical settings and examine its performance under real-world constraints.

In sum, the findings from this study suggest that MBAT is a promising and effective advancement in the adaptive testing toolkit. By fully integrating parameter uncertainty into both trait estimation and item selection, MBAT is capable of enhancing the reliability of multidimensional trait estimates and, therefore, the validity of test score interpretations.

## Data Availability

All materials referred to throughout this manuscript have been made available on the project’s Open Science Framework (OSF) page: https://osf.io/43nxm/overview?view_only=034e10d87b9b4c31a898b016b581a256. Other materials will be provided to interested researchers upon request.

## Code availability

The code required to run the different approaches referred to in the manuscript is available on the project’s Open Science Framework (OSF) page: https://osf.io/43nxm/overview?view_only=034e10d87b9b4c31a898b016b581a256
